# Comprehensive Landscape of Diagnostic, Prognostic and Predictive Biomarkers in Colorectal Cancer: From Genomics to Multi-Omics Integration in Precision Medicine

**DOI:** 10.3390/jpm16010048

**Published:** 2026-01-12

**Authors:** Alfonso Agüera-Sánchez, Emilio Peña-Ros, Irene Martínez-Martínez, Francisco García-Molina

**Affiliations:** 1 Department of Pathology, Hospital General Universitario Reina Sofía, 30003 Murcia, Spain; pacogm@um.es; 2 Faculty of Medicine, Universidad de Murcia, 30120 Murcia, Spain; immlgi@um.es; 3 Department of General and Digestive Surgery, Hospital General Universitario Reina Sofía, 30003 Murcia, Spain; epena@ucam.edu; 4 Faculty of Medicine, Universidad Católica San Antonio de Murcia, 30107 Murcia, Spain; 5 Centro Regional de Hemodonación, Department of Haematology and Oncology, Hospital General Universitario Morales Meseguer, Instituto Murciano de Investigación Biosanitaria (IMIB)-Pascual Parrilla, 30003 Murcia, Spain

**Keywords:** biomarkers, colorectal cancer, genomics, liquid biopsy, precision medicine

## Abstract

Colorectal cancer (CRC) remains one of the leading causes of cancer-related morbidity and mortality worldwide. Despite advances in screening and therapeutic strategies, early detection and individualized treatment remain major challenges. In recent years, an expanding repertoire of biomarkers has emerged, spanning genomic, transcriptomic, proteomic, and metabolomic signatures. Epigenetic features, such as DNA methylation panels, as well as non-coding RNAs and the gut microbiome, hold potential not only for improving early diagnosis but also for refining prognosis and predicting therapeutic responses within the framework of precision oncology. This narrative review provides an updated, integrative overview of CRC diagnostic, prognostic, and predictive biomarkers. We distinguish established markers already in clinical practice, such as *RAS* and *BRAF* mutations, *HER2* amplification, microsatellite instability/mismatch repair deficiency (MSI/dMMR), and widely investigated molecular alterations including *TP53* mutations and immune-checkpoint-related markers, from novel biomarkers with growing translational potential. We also discuss the implementation challenges of these biomarkers in clinical practice, including issues related to validation, standardization, and cost-effectiveness, as well as the multi-modal approach for the development of composite diagnostic panels.

## 1. Introduction

Colorectal cancer (CRC) is a major global health concern. In 2020, approximately 1.93 million new cases of CRC were diagnosed worldwide, with approximately 0.94 million CRC-related deaths, ranking as the third most commonly diagnosed malignancy and the second leading cause of cancer mortality globally [1,2]. Despite the implementation of screening programs that have modestly improved early detection and reduced mortality, a substantial proportion of patients are still diagnosed at advanced stages of disease [3,4]. This late presentation underscores the urgent need for more effective approaches in CRC management, driving intense interest in molecular biomarkers as tools to enhance early diagnosis, guide treatment, and improve outcomes.

Molecular biomarkers have become integral to the clinical management of CRC by influencing multiple aspects of care, including early detection, risk stratification, and personalized therapy selection [5]. Several biomarkers have already been incorporated into standard practice. For instance, genomic profiling via polymerase chain reaction (PCR) or Next-Generation Sequencing (NGS) of *RAS* (*KRAS*/*NRAS*) or *BRAF* is routinely performed in metastatic CRC to guide the use of anti-epidermal growth factor receptor (EGFR) monoclonal antibody therapies. It is crucial to understand that activating *RAS* mutations confer resistance not merely by association but because they constitutively activate the MAPK signaling pathway downstream of EGFR, rendering the upstream receptor blockade biologically ineffective. Similarly, *BRAF*^V600E^ mutations are associated with a poor prognosis and a reduced likelihood of response to anti-EGFR therapy [6]. Evaluation of DNA mismatch repair (MMR) status, typically assessed via immunohistochemistry (IHC) for MMR proteins (MLH1, MSH2, MSH6, PMS2) or PCR for microsatellite instability, is now standard of care. CRC patients with MSI-high (dMMR) tumors are eligible for immune checkpoint inhibitor therapy [6]. While initially approved for metastatic disease, immune checkpoint inhibitors (ICIs) are now being explored in the neoadjuvant setting, where they can substantially reduce tumor burden before surgery and facilitate higher rates of organ preservation and complete pathological response [7]. However, only a minority of CRC patients currently derive significant benefit from such biomarker-driven treatments, highlighting the need to discover new biomarkers that can broaden the impact of precision medicine in CRC management.

Recent technological advances have accelerated the expansion of the CRC biomarker landscape. Next-generation sequencing and high-throughput genomic profiling have enabled the identification of novel genetic and epigenetic markers of CRC, and liquid biopsy approaches are opening avenues for non-invasive diagnostics [5,8]. In particular, circulating tumor DNA (ctDNA) assays and other blood-based biomarkers have shown promise for early cancer detection and for real-time monitoring of minimal residual disease (MRD), offering a potential means to detect recurrence or treatment response before clinical or radiologic changes become apparent [5,9,10,11]. Additionally, emerging biomarker research has begun to explore new categories of molecular indicators, such as gene expression signatures (including consensus molecular subtypes), non-coding RNAs (e.g., tsRNAs, miRNAs, and lncRNAs), and oncogenic gene fusions, which may provide further prognostic information and therapeutic targets in CRC [5,12,13,14,15,16,17]. Collectively, these developments suggest that the integration of innovative biomarkers could significantly improve the early diagnosis of CRC and refine patient-specific treatment strategies.

In this narrative review, we provide an updated overview of the role of molecular biomarkers in colorectal cancer, with a primary focus on diagnostic biomarkers and additional coverage of prognostic, predictive, and therapeutic-response biomarkers. The article is structured as follows: first, we discuss current and emerging diagnostic biomarkers for CRC, including those used in screening and early detection. We then examine key prognostic biomarkers that inform disease outcomes and risk stratification. Next, we review predictive biomarkers that guide therapy selection and personalized treatment—for example, markers predicting response to targeted therapies or immunotherapies. We further address biomarkers used for monitoring therapeutic response and MRD during and after treatment. Finally, we highlight future perspectives and research directions in the field of CRC biomarkers and conclude with a summary of the clinical implications of these advances.

To gather relevant evidence for these topics, a comprehensive literature search was performed using PubMed, Scholar, Web of Science, and Scopus databases, covering mainly publications within the last five years (2020–2025). The search strategy included combinations of the following keywords: “colorectal cancer”, “biomarkers”, “genomics”, “liquid biopsy”, “ctDNA”, “multi-omics”, “transcriptomics”, and “precision medicine”. We prioritized systematic reviews, meta-analyses, and results from major randomized clinical trials, alongside seminal historical papers establishing current guidelines. Articles were selected based on their relevance to clinical practice and translational potential.

## 2. Diagnostic Biomarkers in Colorectal Cancer

**Figure 1:**
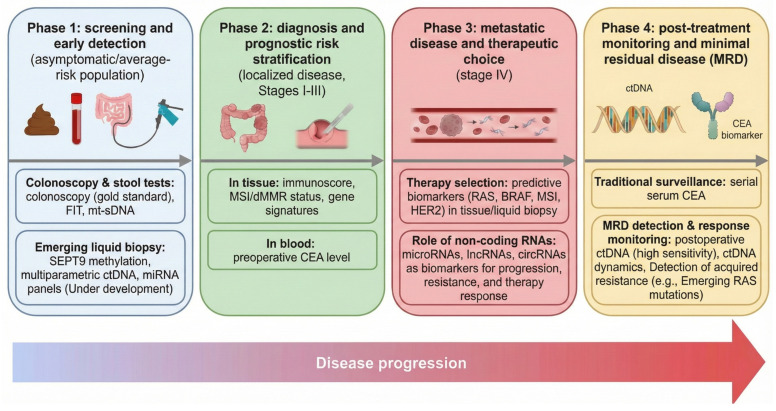
Integration of multimodal biomarkers across the clinical continuum of colorectal cancer (CRC). This scheme summarizes the application of biomarkers in different clinical scenarios, as reviewed in Section 2, Section 3 and Section 4. In the screening phase (blue chart), stool-based tests (FIT, mt-sDNA) and colonoscopy are used, whereas liquid biopsy approaches (ctDNA, miRNAs) are being explored for early detection. In localized disease (green chart), tissue profiling (MSI, Immunoscore) and serum CEA refine prognosis. In the metastatic setting (red chart), genomic predictive markers such as *RAS*, *BRAF*, MSI, and *HER2* guide initial therapy selection. Finally, serial liquid biopsy techniques (e.g., tumor ctDNA and MRD detection; yellow chart) enable dynamic response monitoring and early detection of resistance mechanisms.

Early detection of colorectal cancer (CRC) is critical for improving patient outcomes. Colonoscopy remains the gold standard for CRC diagnosis and the removal of precursor lesions (Figure 1) [18]. However, a substantial proportion of young patients are still diagnosed at advanced stages despite existing screening programs [19,20]. This has spurred intensive research into non-invasive and cost-effective diagnostic biomarkers that complement or enhance current screening methods by enabling early detection with greater sensitivity and specificity, thereby facilitating timely intervention and improved survival [21,22,23]. In this section, we review key categories of diagnostic biomarkers in CRC, including standard of care screening tools such as fecal occult blood testing (FOBT) and fecal immunochemical test (FIT), but also novel fecal and blood-based assays such as DNA methylation markers, circulating tumor DNA, exosomal biomarkers, and microbiome analysis, along with their performance characteristics, clinical utility, and potential roles in early detection (Table 1).

### 2.1. Traditional Serum Tumor Markers

Carcinoembryonic antigen (CEA) and carbohydrate antigen 19-9 (CA19-9) are classic blood-based tumor markers measured in serum using standard immunoassays (e.g., ELISA or chemiluminescence). However, neither marker is recommended on their own as a primary screening tool for average-risk populations. CEA is a glycoprotein normally expressed during fetal development, and it can be elevated in the blood of patients with CRC as well as in several other malignancies or benign conditions [24]. Due to limited sensitivity and specificity for early detection, as well as weak association with tumor differentiation, diameter, and staging, the main clinical utility of CEA is post-treatment surveillance (see, Section 4.4) [25,26]. An elevated preoperative CEA level also has prognostic significance, correlating with a higher risk of disease recurrence and worse survival (discussed further in Section 3) [27]. CA19-9, a sialylated Lewis antigen, is primarily associated with pancreaticobiliary cancers but can also be elevated in advanced CRC [28]. However, CA19-9 is even less sensitive and specific than CEA for early CRC and is not used for screening; at most, it may have adjunct prognostic and predictive value in metastatic disease [29]. In summary, CEA—and to a lesser extent, CA19-9—are important as surveillance biomarkers and as part of multi-marker panels; however, they are insufficient for reliable CRC early detection.

### 2.2. DNA-Based Biomarkers in Stool and Blood

Requiring specific pre-analytical stool stabilization buffers, fecal occult blood testing (FOBT) and fecal immunochemical test (FIT) are two examples of longstanding non-molecular screening tools that detect blood in stool [30,31]. FIT is widely used and has higher sensitivity for CRC than older guaiac-based tests, but it can yield false-positives from non-tumor bleeding and has limited sensitivity for advanced adenomas [32]. To improve upon this technique, multi-target stool DNA panels that combine hemoglobin detection with tumor-derived DNA alterations (e.g., aberrantly methylated genes and mutated oncogenes) have been developed [22]. A prime example is the FDA-approved multitarget stool DNA (mt-sDNA) test (Cologuard), which assays methylated *BMP3/NDRG4*, mutant *KRAS*, and hemoglobin. This test achieves a CRC sensitivity of ~92%—substantially higher than FIT—but at the cost of more false positives (specificity ~86%) and a much higher cost per test [33]. Indeed, while U.S. guidelines endorse stool DNA testing as a “second-tier” option, it is not widely adopted outside the USA due to its lower specificity and cost (up to $600 per test, whereas a single FIT kit typically costs on the order of $20–$30) [34,35]. Notably, an improved second-generation stool DNA assay was recently reported to detect CRC with 93.9% sensitivity (43.4% for advanced precancerous lesions), marking significant progress in stool-based detection [33].

In parallel, blood-based DNA markers have emerged as attractive minimally invasive diagnostic tools. The SEPT9 DNA methylation blood test was the first plasma biomarker approved for CRC screening [36]. Methylated *SEPT9* in cell-free DNA (cfDNA) can be detected in a fraction of CRC patients’ plasma; however, its sensitivity in early stage disease is modest (~60–70%), and its overall performance is inferior to that of FIT in head-to-head comparisons, limiting its adoption in population screening [37,38,39]. Recently, advances in circulating tumor DNA (ctDNA) technology have enabled the detection of tumor-derived mutations or methylation in the blood with improved sensitivity. For example, a next-generation cfDNA assay reported ~83% sensitivity for detecting known CRC (though only ~13% for precancerous adenomas) [40]. Such blood-based tests offer convenience and high specificity, but the main challenge remains the sensitivity in asymptomatic early stage CRC, when the concentration of tumor DNA in blood is very low. Ongoing efforts are exploring panel-based ctDNA assays and multi-omics approaches—integrating DNA mutations, methylation, and possibly protein markers—to enhance sensitivity for early lesions [41,42]. Indeed, liquid biopsy approaches are rapidly evolving and hold promise for real-time early cancer detection, although they are not yet recommended for routine population screening and are pending further validation [43,44,45]. For example, a recent study by Brenne et al. in a pre-diagnostic screening population—the HUNT study—found that while specific methylation markers were detectable, the overall sensitivity for identifying future CRC cases was limited to 43% [44], highlighting the challenge of maintaining high diagnostic performance outside of clinical case–control settings.

Overall, colonoscopy remains the cornerstone of CRC screening due to its superior sensitivity and the preventive benefit of removing premalignant lesions, whereas stool-based tests such as FIT are widely implemented as non-invasive, cost-effective tools in average-risk population screening. Nonetheless, the trajectory is encouraging: ctDNA analysis, initially confined to metastatic disease genotyping, is now being tested for screening and early detection purposes, potentially as part of risk-adapted screening strategies or in conjunction with existing modalities.

### 2.3. RNA-Based Biomarkers

The evolving landscape of CRC biomarkers has expanded to include a vast network of regulatory non-coding RNAs that play fundamental roles in tumor biology. Both miRNAs and lncRNAs have demonstrated significant promise as biomarkers for CRC that can be detected in serum or plasma, offering improvements in early detection, prognostication, and therapy personalization.

Circulating microRNAs (miRNAs) are abundantly released by tumors into the bloodstream, often encapsulated in exosomes or other extracellular vesicles, and many show differential expression between patients with CRC and healthy individuals [46]. Notably, while individual miRNA biomarkers often lack sufficient sensitivity or specificity on their own, composite miRNA signatures or the combination of miRNAs with other markers can significantly improve diagnostic performance [47]. For instance, miR-21, miR-23, and miR-92a are two oncogenic miRNAs that have been consistently reported to be elevated in the plasma of patients with colorectal cancer (CRC), reflecting their active involvement in tumor initiation and progression [48,49]. These molecules can be detected even in early stage disease, highlighting their potential value as minimally invasive biomarkers for early diagnosis [50,51]. Building upon this concept, a recent study employing advanced machine learning algorithms identified a distinctive four-miRNA signature derived from tumor-secreted exosomes—miR-23a-3p, miR-92a-3p, miR-125a-3p, and miR-150-5p—that achieved high accuracy in distinguishing CRC patients from healthy individuals at all stages [52]. More generally, numerous exosomal miRNAs have been proposed as non-invasive diagnostic markers. For example, elevated exosomal levels of miR-17-5p, miR-18a/b, and miR-181a-5p have been observed in CRC patients relative to healthy individuals [53]. Likewise, reduced circulating exosomal miR-150-5p and miR-99b-5p have been reported as potential early indicators of CRC [54]. This combination of exosomal miRNAs not only underscores the diagnostic potential of circulating RNA profiles but also illustrates how integrating computational approaches with liquid biopsy analyses can refine the molecular characterization of CRC and support the development of next-generation, non-invasive screening strategies [55].

Long non-coding RNAs (lncRNAs) are >200 nucleotide transcripts with no coding potential that have likewise emerged as critical players in CRC, with numerous studies since 2020 demonstrating their value as molecular biomarkers [56]. LncRNAs are relatively stable circulating molecules that play a role in regulating gene expression through various mechanisms, including chromatin modulation, acting as competitive endogenous RNAs that "sponge" miRNAs, influencing mRNA stability, and interacting with signaling proteins, all of which can contribute to tumor initiation and progression [57,58]. Consequently, lncRNAs are being actively investigated as diagnostic, prognostic, and predictive biomarkers in colorectal cancer. Many patients with CRC exhibit aberrant lncRNA expression profiles in both tumor tissues and circulating biofluids. In general, oncogenic lncRNAs tend to be upregulated in CRC and are associated with more aggressive disease features and poorer clinical outcomes. For instance, the Colorectal Cancer-Associated Transcript family (CCAT lncRNAs) is markedly overexpressed in colorectal tumors, and this overexpression correlates with increased tumor invasiveness and a higher incidence of lymph node metastasis [59]. Another example is lncRNA RPPH1, whose high expression in CRC tissues has been linked to advanced tumor stage and poorer patient survival [5], as well as CASC21, which was shown to promote colorectal carcinogenesis by enhancing cancer cell proliferation, migration, and epithelial-to-mesenchymal transition (EMT) [60]. Mechanistically, CASC21 functions as a competing endogenous RNA that recruits transcriptional regulators and “sponges” a tumor-suppressor miRNA (miR-485-5p), thereby upregulating pro-oncogenic targets that fuel CRC progression. Another lncRNA, XIST, has been found to modulate both tumor growth and drug sensitivity in CRC: the XIST/miR-125b-2-3p axis regulates cancer cell proliferation and also contributes to chemotherapeutic resistance by controlling the WEE1 signaling pathway [61]. In general, lncRNAs can promote metastasis as seen with CASC21 and XIST, which both drive colorectal tumor invasion and dissemination by repressing anti-metastatic miRNAs and inducing EMT [60,61]. Importantly, lncRNAs are not only prognostic indicators but may also predict therapeutic responses, as recent studies have identified several lncRNAs that influence CRC patients’ responses to targeted therapies. High expression of certain lncRNAs, such as MIR100HG and UCA1, has been linked to resistance mechanisms and poor response to EGFR inhibitors like cetuximab and panitumumab [62,63,64]. Moreover, integrative analyses have begun to connect lncRNA signatures with the molecular subtypes of CRC and the tumor immune microenvironment [65], further underscoring the biomarker potential of lncRNAs in the era of precision oncology.

### 2.4. Other Emerging Biomarkers and Combined Approaches

Exosome-based protein markers are another novel approach. Tumor-derived exosomes carry proteomic cargo reflective of their cell of origin, including proteins involved in invasion and metastasis [66]. Their appeal lies in exosomes’ stability and enrichment in blood: the lipid bilayer vesicles protect their RNA/protein cargo from degradation, enabling reliable detection of the biomarkers. One example is exosomal MMP-14 (matrix metalloproteinase-14), which was recently identified as a potential early CRC biomarker detectable in patient plasma. Preliminary data suggest that it may even flag advanced adenomas, although larger validation studies are needed [67].

Another intriguing avenue is the gut microbiome, which can be analyzed using 16S rRNA gene sequencing or shotgun metagenomics of stool samples. It is now well-recognized that certain bacterial species are associated with colorectal carcinogenesis. For example, *Fusobacterium nucleatum*, a bacterium enriched in some CRC patients, can promote colorectal tumor growth through Wnt/β-catenin signaling activation [68]. Another example is *Porphyromonas gingivalis*, a gram-negative anaerobic bacterium considered one of the major pathogens responsible for periodontitis [69]. The overabundance of these microorganisms, among other microbial shifts in stool, has been proposed as a biomarker to flag individuals at risk for CRC [5,68]. While microbial biomarkers are still in the exploratory stage, they exemplify the broadening scope of research on non-traditional diagnostic indicators.

Certain fecal protein assays have also been investigated: fecal M2-pyruvate kinase (M2-PK), an enzyme upregulated in cancer metabolism, can be measured in stool and has demonstrated moderate sensitivity for CRC in pilot studies [70]. However, like FIT, these protein markers can be influenced by non-cancer conditions and have not supplanted established screening tests.

The combination of different types of biomarkers can harness their complementary strengths. A striking illustration is a recent report on tRNA-derived small RNA fragments (tsRNAs), a novel class of non-coding RNAs: plasma levels of a 5′-tRF-Glycine (tRF-Gly-GCC) were able to distinguish CRC cases with an area under the curve (AUC) of ~0.88, and when this tsRNA was combined with classic serum tumor markers (CEA and CA19-9), the diagnostic sensitivity reached 86% with 84% specificity [71]. Similarly, pairing multiple tsRNAs (e.g., an upregulated Ala-derived tRF and a downregulated Tyr-derived tRF) with CEA achieved superior accuracy for CRC detection compared to any single marker alone [72]. Given that no single biomarker is perfect, a clear trend is the move toward multi-analyte tests and precision diagnostic models that include integrative biomarker panels in combination with existing methods, such as colonoscopy and FIT.

## 3. Prognostic Biomarkers in Colorectal Cancer

Prognostic biomarkers provide information about the likely course or outcome of the disease, such as risk of recurrence or overall survival, independent of specific therapies. In CRC, prognosis is chiefly determined by clinicopathological factors, especially the tumor stage at diagnosis; however, a growing array of molecular biomarkers has been identified that refine risk stratification among patients with similar clinicopathological features. This section discusses important prognostic biomarkers in CRC, spanning both tumor-intrinsic molecular features and circulating biomarkers, and highlights how multi-omics integration enhances prognostic assessments.

### 3.1. Tumor-Based Prognostic Factors

Tumor-infiltrating lymphocytes (TILs) represent an essential component of the antitumor immune response, and their presence in the colorectal cancer microenvironment has established itself as an immunological biomarker of great prognostic relevance. Numerous studies have shown that a high density of TILs in colorectal tumor tissue is associated with better survival and a lower risk of relapse, making lymphocyte infiltration an independent favorable indicator [73,74]. The quantification of this immune infiltration has given rise to the concept of Immunoscore, a validated scoring system that assesses the density of T lymphocytes (total CD3+ and cytotoxic CD8+) both in the center of the tumor and in its invasive margin [75,76]. Patients with a high Immunoscore (immunologically “hot” tumors) have a significantly lower risk of recurrence than those with a low Immunoscore, even outperforming conventional TNM staging in prognostic power [77]. An international validation study confirmed that the Immunoscore can improve risk stratification in stage I–III colon cancer, guiding the selection of adjuvant treatment in patients with stage II–III colon cancer [78]. Overall, these instances demonstrate that analyzing the molecular and immune characteristics of tumors can enhance prognostic accuracy beyond the capabilities of standard histopathological methods.

DNA mismatch repair (MMR) deficiency, reflected by high-level microsatellite instability (MSI-H), also has important prognostic implications. Approximately 15% of early stage (I–III) CRCs are MSI-H due to sporadic or hereditary (Lynch syndrome) MMR defects, whereas only ~5% of metastatic CRCs are MSI-H [27]. MSI-H tumors tend to be biologically distinct, with abundant lymphocytic infiltration and a suppressed WNT pathway [79], in contradistinction to what has been established in MSS or “immune-cold” tumors [80,81]. Overall, MSI-H or dMMR status is associated with a better stage-adjusted prognosis, although this remains controversial for stage I [27,82]. Patients with stage II MSI-H colon cancers, for example, have excellent outcomes (5-year overall survival ~80–85%) and derive little benefit from adjuvant chemotherapy in absence of any high-risk feature, which is why guidelines consider stage II MSI-H itself a low-risk feature where chemotherapy can be omitted [83,84]. In stage III disease, large pooled analyses indicate that MSI-H tumors might have slightly improved survival compared to MSS when treated with surgery and adjuvant chemotherapy [85], although recent meta-analyses have yielded mixed conclusions [86]. In the metastatic setting, prior to immunotherapy availability, MSI-H status did not confer a survival advantage (and may even have been associated with shorter survival in some series) [87]. Notably, MSI-H mCRC patients can now benefit from immunotherapies, and dual checkpoint inhibition has recently demonstrated a massive improvement in progression-free survival compared to chemotherapy (single line), fundamentally transforming outcomes for this subgroup [88]. Overall, MSI/dMMR is considered a favorable prognostic factor in localized CRC (particularly stage II), whereas in metastatic disease MSI-H by itself does not improve prognosis in the absence of immunotherapy. Beyond survival, MSI status is also used to identify patients who should undergo genetic evaluation for Lynch syndrome, as universal MMR/MSI testing is recommended for all new CRC cases.

Integrating TILs density with other immunological biomarkers, such as MSI status, provides more accurate prognostic stratification in colorectal cancer. A recent meta-analysis involving more than 14,000 patients showed that combining MSI status with TIL infiltration allows for the definition of four tumor subtypes with different prognoses [89]. In this study, MSI-H tumors with high TIL infiltrate showed the best prognosis, closely followed by MSS with high TIL; conversely, poor lymphocyte infiltration (low-TIL) was associated with significantly worse survival regardless of MSI status.

Among the best-established molecular prognostic markers in CRC are mutations in *KRAS/NRAS* and *BRAF*. *BRAF*^V600E^ mutations, present in ~8–10% of CRCs, are associated with a distinctive clinical phenotype (predilection for proximal colon location, aggressive behavior, and frequent peritoneal or distant lymph node metastases) and confer an unfavorable prognosis [90]. Multiple retrospective studies and meta-analyses have identified *BRAF*^V600E^ as an independent predictor of poor survival. In metastatic CRC, *BRAF*^V600E^-mutant patients have historically had markedly shorter overall survival (median OS roughly half that of *BRAF*-wildtype patients), reflecting the aggressive biology and limited effectiveness of standard therapies in this subset [6,91]. By contrast, non-V600E *BRAF* mutations (which occur in a smaller fraction of CRCs) do not seem to carry the same adverse prognostic weight and may portend better outcomes than V600E mutations [92]. *KRAS* or *NRAS* mutations (found in ~50% of CRCs) have a more nuanced prognostic impact that depends heavily on the specific amino acid substitution: Koulouridi et al. revealed that *KRAS*^G12D^ mutations (the most common subtype, ~33%) were significantly correlated with better overall survival (*p* = 0.04) compared to other G12 mutations [93]. In contrast, *KRAS*^G12C^ mutations (~4.8%) were associated with a worse prognosis, presenting shorter progression-free survival (PFS) and overall survival rates than other subtypes. This suggests that distinct *KRAS* variants drive different biological behaviors, with G12D and G12C leading to better and worse outcomes, respectively. Overall, although *RAS* mutations do not serve as a prognostic indicator as robustly as *BRAF*^V600E^, the occurrence of ctDNA *KRAS* mutations following surgery has been associated with worse disease-free survival, as detailed in Section 3.2.

Comprehensive transcriptomic and integrative analyses have defined some intrinsic subtypes of CRC that correlate with prognosis. The Consensus Molecular Subtypes (CMS) classification stratifies CRC into four molecular subtypes: CMS1 (MSI-Immune), CMS2 (Canonical), CMS3 (Metabolic), and CMS4 (Mesenchymal) [6,92]. These subtypes have distinct biology and outcome patterns that respond to unique gene expression signatures. CMS4 tumors, characterized by TGF-β activation and stromal infiltration, display the worst outcomes; notably, one study reported a 5-year relapse-free survival of only ~60% for CMS4 patients compared to ~75% for CMS1 and CMS2/3 patients [94]. CMS1 tumors (largely MSI-H with strong immune activation) often have a better prognosis in early stages (owing to immune-mediated tumor control) but paradoxically may respond less favorably to conventional chemotherapy in advanced disease. CMS2 and CMS3 have intermediate prognoses. Although CMS subtyping is not yet used in routine practice, it provides a multi-omics framework integrating genomic, epigenomic, and transcriptomic data that improves our understanding of CRC heterogeneity and could inform future prognostic tools. In parallel, specific gene expression-based assays have been developed for prognostic purposes. For example, a 12-gene recurrence score (Oncotype DX Colon) was shown to stratify recurrence risk in stage II colon cancer, identifying a subset of patients with low scores who had <10% risk of recurrence at 3 years with surgery alone [95]. Another clinically evaluated panel is ColoPrint (18-gene signature), which also classifies early stage CRC into low vs. high recurrence risk groups, helping to identify high-risk stage II patients who may benefit from adjuvant chemotherapy [96].

### 3.2. Circulating Prognostic Biomarkers

Balta et al. found that patients with CEA > 5 ng/mL before surgery had significantly worse disease-free and overall survival than those with normal CEA levels. In particular, high CEA levels were associated with a hazard ratio (HR) of ~2.3 for death or recurrence [97]. Therefore, elevated preoperative serum CEA levels are an adverse prognostic factor for CRC. CA19-9 is not routinely measured for prognostic stratification in colon cancer; however, in patients with metastatic or locally advanced CRC with elevated CA19-9 levels, very high levels may portend worse outcomes and suggest pancreatic involvement or an alternate pathology [98]. Interestingly, recent research has proposed other serum markers such as CA-125 or CA-242 as potentially stronger prognostic indicators than CEA in CRC [99], though these are not widely used. Overall, CEA remains the most utilized serum prognostic marker due to its widespread availability and correlation with tumor burden, and guidelines recommend incorporating CEA into baseline risk assessment and follow-up [100]. However, CEA lacks specificity, and novel blood-based markers are being explored to complement it.

Circulating Tumor Cells (CTCs) represent cancer cells that detach from the primary tumor. Their presence highlights the concept of the “tumoral niche,” where CTCs prepare distant sites for colonization [101]. In general, the presence of CTCs in peripheral blood has been associated with worse outcomes. For example, using the CellSearch^®^ platform, an FDA-cleared immunomagnetic method for CTC enumeration, detection of ≥1 CTC/7.5 mL blood preoperatively has been shown in some studies to be an independent predictor of shorter relapse-free survival [101,102]. In metastatic CRC, a cutoff of ≥3 CTCs/7.5 mL is often used, and patients above this threshold have significantly poorer progression-free and overall survival than those with fewer CTCs [101,102]. However, results across studies have not been uniformly consistent, and some trials did not find postoperative CTC counts to be prognostically significant [27]. Moreover, there is heterogeneity in CTC phenotypes (epithelial vs. mesenchymal CTCs), and technical differences in assays [27,103]. Despite these challenges, CTCs are a promising prognostic tool, particularly for advanced CRC. Yu et al. identified a subset of CTCs expressing cell-surface vimentin (CSV-CTCs) and found that patients with ≥3 CSV-CTCs had a significantly higher risk of disease progression (HR ~3.8) [103]. As technology improves, CTC characterization (beyond just counting) may yield additional prognostic and predictive insights, such as identifying specific mutations or markers of EMT and stemness in CTCs that could inform treatment.

One of the most transformative recent advancements in CRC prognostication is the use of circulating tumor DNA (ctDNA) for MRD detection. After curative-intent surgery (or adjuvant therapy), a fraction of patients harbor occult cancer cells that may eventually cause relapse. ctDNA assays can detect minute amounts of tumor-derived DNA in plasma, essentially serving as a “liquid biopsy” for residual disease, and thereby being a powerful predictor of recurrence before any clinical signs appear [104,105]. In a large cohort study of 1039 patients, the presence of ctDNA 4–8 weeks post-surgery was associated with a >10-fold higher risk of relapse compared to ctDNA-negative patients [106]. Prospective trials, such as GALAXY, have shown that ctDNA-positive patients after curative surgery have markedly worse 18-month disease-free survival (~38%) than ctDNA-negative patients (~91%), even when imaging shows no evidence of disease [107]. Similarly, Tie et al. evaluated stage II colon cancer patients and found that those with ctDNA detectable in plasma after surgery—and who did not receive chemotherapy—had a very high recurrence rate (~79% recurrence at 2–3 years), whereas ctDNA-negative patients had a much lower recurrence rate [108]. Furthermore, the presence of ctDNA after adjuvant chemotherapy was associated with a dramatically reduced recurrence-free survival (HR 11). These findings were supported by a recent systematic review and meta-analysis of over 2400 patients, demonstrating that postoperative ctDNA positivity is the strongest independent predictor of impaired recurrence-free survival in stage III CRC [109].

However, to ensure clinical safety, the choice of assay is critical. Current strategies fall into two main categories: tumor-informed assays (tracking patient-specific mutations identified from tissue) and tumor-agnostic (or tumor-naïve) assays (using fixed panels of common mutations/methylation). Tumor-informed approaches generally demonstrate higher sensitivity for detecting MRD, particularly in low-shedding scenarios, with a reported Hazard Ratio for recurrence prediction significantly higher than tumor-agnostic methods in comparative meta-analyses [110]. Regarding timing, blood collection is typically recommended 4–8 weeks post-surgery to minimize false positives from trauma-induced cell-free DNA, a protocol validated in major prospective trials, such as GALAXY [106]. Major limitations persist, specifically false negatives in “low-shedding” tumors (e.g., mucinous histology or peritoneal metastases) and the challenge of “ctDNA-positive/imaging-negative” patients, for whom the optimal intervention window remains under investigation [104].

### 3.3. Integrative and Multi-Omics Prognostic Approaches

As evidenced above, a wide array of factors, from gene mutations and expression signatures to immune contexture and circulating biomarkers, can influence CRC prognosis. Increasingly, multi-omics integration is being explored to build composite prognostic models that combine genomic data (like *KRAS*/*BRAF* mutations and MSI status), transcriptomic subtypes (CMS or immune signatures), and even microbiome or metabolomic profiles to refine risk groups [27,111], outperforming single-omic approaches. Machine learning and AI methods have also been employed to analyze these complex datasets and identify novel prognostic patterns or biomarker combinations [111,112]. In the PROMISE study, a multi-cancer liquid biopsy classifier combining cfDNA methylation and protein biomarkers achieved higher cancer detection sensitivity (75.1%) at the same specificity (98.8%) compared to a methylation-only model [113]. Similarly, an integrated prognostic signature in CRC built on transcriptomic and other omics features, the ICDRS, showed time-dependent AUCs > 0.90 for 1–3 year survival [114]. One recent multi-omics study identified an eight-metabolite panel derived from gut microbiome activity that, when combined with clinical factors, achieved an AUC > 0.90 for distinguishing high-risk from low-risk CRC patients [115]. Another approach uses radiogenomics, which integrates imaging features with genomic markers to improve prognostication beyond either modality alone [116]. The consensus from emerging literature is that no single biomarker can capture the full complexity of tumor behavior; therefore, holistic models incorporating diverse data (genetic, epigenetic, immunologic, etc.) are likely to provide the most robust prognostic stratification. Moving forward, large-scale collaborations and databases (e.g., The Cancer Genome Atlas and international consortia) will continue to be leveraged to validate prognostic multi-omics signatures, with the ultimate goal of translating these into clinically useful prognostic tests that can guide the intensity of surveillance and adjunctive therapy for CRC patients.

## 4. Predictive Biomarkers: Treatment Selection and Therapeutic Response Monitoring

**Figure 2:**
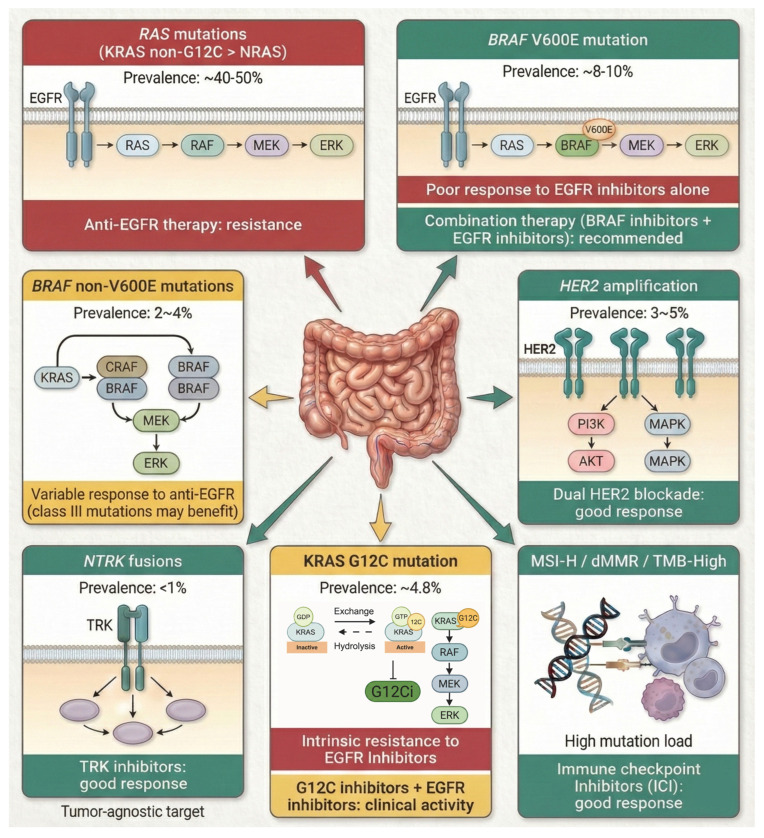
Landscape of predictive biomarkers and therapeutic implications in metastatic colorectal cancer (mCRC). This diagram depicts the approximate prevalence of key actionable alterations with their associated signaling pathways and targeted treatment outcomes. Color-coded indicators reflect clinical response based on current evidence: Green indicates sensitivity (e.g., ICI for dMMR/MSI-H, dual blockade for HER2+, combination therapy for BRAF V600E); Red denotes poor response/resistance (e.g., anti-EGFR in *RAS*/*BRAF*-mutants); and Yellow represents uncertain response. ***Abbreviations***: *G12Ci*: *KRAS G12C inhibitors*; *ICI: Immune Checkpoint Inhibitors*; *TMB*: *Tumor Mutational Burden*.

**Figure 3:**
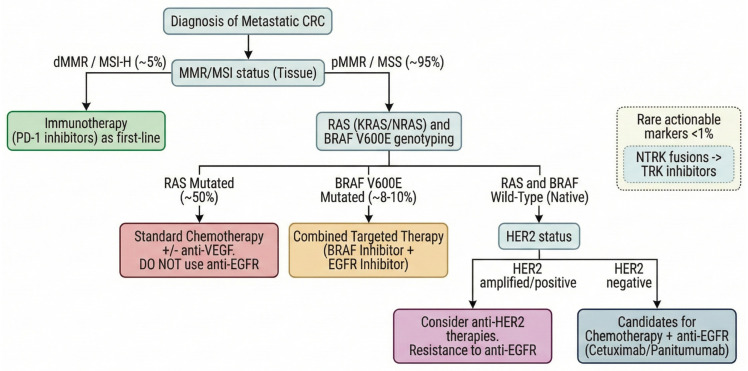
Practical algorithm for integrating predictive biomarkers into the first-line management of metastatic colorectal cancer (mCRC). This flowchart illustrates the hierarchy of essential molecular tests for personalized therapy selection. Microsatellite instability (MSI/dMMR) status is the first critical determinant, identifying patients eligible for immunotherapy. For microsatellite-stable (MSS) tumors, the minimum mandatory panel must include mutational status of *RAS* (at least *KRAS* and *NRAS* exons 2, 3, and 4) and *BRAF*^V600E^, fundamental to defining eligibility for anti-EGFR therapies or *BRAF*-targeted combinations, respectively. In patients without *RAS*/*BRAF* mutations, an extended panel with *HER2* assessment could identify an additional subgroup that may benefit from *HER2*-targeted therapies instead of EGFR inhibitors. Rare markers like *NTRK* fusions and potentially *MET* amplification or *KRAS*^G12C^ specific mutations offer tissue-agnostic therapeutic options.

Predictive biomarkers are indicators that forecast a tumor’s likely response (or resistance) to a particular therapy. In CRC, the paradigm of predictive biomarkers is well established in the metastatic setting; for example, *RAS* mutations predict the lack of benefit from anti-EGFR monoclonal antibodies, and MSI predicts the benefit from immunotherapy (Figure 2). This section reviews key predictive biomarkers that guide treatment selection in CRC, including markers for targeted therapies and immunotherapies with a stepwise molecular algorithm for biomarker testing in metastatic CRC (Figure 3). We organized the discussion by therapeutic context: (1) biomarkers guiding immune checkpoint inhibitor therapy, (2) biomarkers guiding the use of EGFR-targeted and other related targets, (3) less frequent targetable mutations (*HER2*, *NTRK*, etc.), and (4) emerging circulating biomarkers for real-time monitoring of therapeutic response and resistance.

### 4.1. Microsatellite Instability and Immune Checkpoint Inhibitor (ICI) Therapy

The most important predictive biomarker for immunotherapy in metastatic CRC is the MSI/dMMR status. As discussed in Section 3, MSI-high tumors have an elevated mutation load and neoantigen burden, which makes them susceptible to immune checkpoint blockade. The phase II KEYNOTE-164 trial led to the approval of pembrolizumab (anti-PD-1) for MSI-H/dMMR mCRC [117]. In phase III KEYNOTE-177 trial, first-line pembrolizumab doubled the median progression-free survival (16.5 vs. 8.2 months) compared to standard chemotherapy in MSI-H mCRC (HR 0.60, *p* < 0.001) [118]. Based on such data, pembrolizumab or nivolumab (±ipilimumab) is now recommended as first-line therapy for metastatic dMMR CRC [119]. In addition, recent studies have demonstrated that ICI could also play a role in unresectable non-metastatic but locally advanced dMMR/MSI-H CRC [120]. Conversely, microsatellite-stable (MSS) CRCs (~95% of metastatic cases) show minimal response to single-agent PD-1/L1 inhibitors; therefore, MSS status predicts a lack of benefit from immunotherapy (unless combined with other strategies) [121]. This stark dichotomy underlines the need for MMR or MSI testing in all patients with newly diagnosed metastatic CRC.

In the neoadjuvant setting, there have been striking case series (and a recent small trial) showing that checkpoint inhibitors can even induce complete responses in localized MSI-H rectal cancer, potentially obviating the need for chemoradiation or surgery in some patients [122,123]. Although these approaches are still investigational, they reinforce MSI as a key predictive marker for immunotherapy across stages. Notably, >50% of MSI-H CRC patients do not respond to PD-1 blockade (primary resistance) or may acquire resistance after an initial response [27]. Research is ongoing to identify additional predictive markers among MSI-H tumors to distinguish responders from non-responders (for example, evaluating tumor mutational burden as a continuous variable, immune gene expression profiles, or specific mutations such as *JAK1/2* or *β2M* associated with immune resistance) [124]. So far, tumor mutational burden (TMB) is correlated with MSI but has not added clear predictive value beyond MSI status in CRC [125]. Additionally, PD-L1 expression has been correlated with overall better prognostic outcomes and may become a reliable predictor in CRC since most MSI-H CRCs are PD-L1 positive, with variable predictive reliability depending on the antibody clone and scoring method used [126,127].

Ongoing trials are exploring whether certain immunoscore or tumor microenvironment features in MSS CRC could predict benefit from emerging immunotherapy combinations—e.g., adding VEGF inhibitors or novel immunomodulators to overcome resistance—[128,129], but such biomarkers remain exploratory.

### 4.2. RAS/RAF/EGFR Pathway Alterations

The discovery that mutations in the *RAS* pathway confer resistance to EGFR-targeted monoclonal antibodies was a watershed for personalized CRC therapy. Cetuximab and panitumumab, which target the EGFR extracellular domain, were initially approved for metastatic CRC without patient selection; however, only a subset of patients responded [130]. It is now firmly established that tumors harboring activating mutations in *KRAS* or *NRAS* exon 2, 3, or 4 do not respond to anti-EGFR therapy, because the downstream signaling is constitutively active [131]. Therefore, current guidelines mandate *RAS* genotyping (of at least *KRAS* and *NRAS*) for all patients being considered for EGFR inhibitor therapy [100,132].

Approximately 50% of mCRC patients have *RAS* mutations and are ineligible for EGFR antibodies [133,134]. Among *RAS* wild-type patients who receive cetuximab or panitumumab, about 50–60% respond or have disease control [135]. Notably, even in *RAS* wild-type cases, acquired resistance to EGFR blockade frequently develops through emergent *RAS* mutations: small subclones with *RAS* mutations are often undetectable at baseline but can be selected under therapy pressure [136]. These emergent mutations can now be detected by ctDNA analysis a few months into therapy. For instance, one study showed that new *RAS* mutations appeared in blood on average 3 months before radiologic progression, and patients in whom such mutations emerged had significantly shorter progression-free and overall survival on anti-EGFR therapy [136]. This has two implications: (1) liquid biopsy can monitor and predict resistance, and (2) *RAS*-mutant clones often fade after stopping EGFR therapy, prompting trials to reintroduce EGFR inhibitors once a patient’s blood tests negative for *RAS* mutations again [137,138]. Indeed, the CRHONOS trial demonstrated in 2023 that in patients who initially responded and then progressed on anti-EGFR therapy, those who had no *RAS*/*BRAF* mutations in ctDNA after a “drug holiday” could benefit from anti-EGFR rechallenge (objective responses ~30%) [138]. Thus, *RAS* mutations are both a negative predictive biomarker (for initial therapy selection) and a tool for dynamic response monitoring.

An additional clinically relevant subset is represented by tumors harboring *KRAS*^G12C^ mutations. Allele-specific KRAS^G12C^ inhibitors have recently shown activity in *KRAS*^G12C^-mutated CRC, particularly when combined with EGFR blockade, although response rates are lower than those observed in non-small cell lung cancer, and acquired resistance frequently emerges through on-target and bypass mechanisms [139,140]. Importantly, there are currently no clinically available pan-KRAS inhibitors capable of targeting all *KRAS*-mutant alleles, and the development of such agents remains an active area of research.

*BRAF*^V600E^, besides being a negative prognostic marker as discussed, is also predictive of poor response to EGFR inhibitors when used alone. *BRAF*^V600E^-mutant mCRCs rarely respond to cetuximab or panitumumab monotherapy, likely because the activated BRAF kinase drives signaling independent of EGFR, and feedback loops upregulate EGFR when BRAF is inhibited [141]. For many years, patients with *BRAF*-mutant mCRC had limited treatment options; however, recent advances have introduced BRAF-targeted therapeutic strategies. The BEACON CRC trial showed that combining a BRAF inhibitor (encorafenib) with an EGFR inhibitor (cetuximab), with or without a MEK inhibitor, significantly improved survival in *BRAF*^V600E^ metastatic CRC compared to standard chemotherapy [142]. Encorafenib + cetuximab is now an approved regimen for these patients, roughly doubling median overall survival (to ~9.3 months) relative to historical controls [143]. Therefore, *BRAF*^V600E^ is a predictive biomarker indicating that a patient should receive targeted therapy (BRAF/EGFR inhibition) rather than EGFR inhibitors alone or conventional treatment. It is worth noting that, unlike melanoma, single-agent BRAF inhibitors are ineffective in CRC (due to EGFR-mediated feedback activation); therefore, a combination approach is critical. Non-V600E *BRAF* mutations do not confer resistance to EGFR therapies and are currently treated as *RAS*/*BRAF* wild types [132]. Ongoing research, such as the BREAKWATER trial, is investigating the use of encorafenib + cetuximab in first-line therapy for *BRAF*-mutant CRC, which may further improve outcomes [144]. In summary, *BRAF*^V600E^ testing is essential at the time of diagnosis of mCRC for both prognostication and to guide the use of the EGFR/BRAF-targeted regimen, which has become the standard of care for this subset.

**Figure 4:**
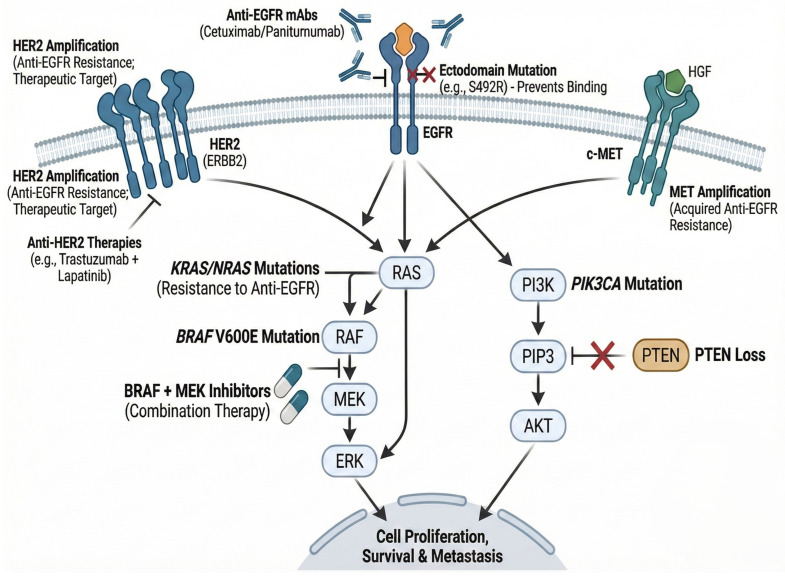
Key signaling pathways and predictive biomarkers in metastatic CRC therapy. This schematic highlights the canonical EGFR-RAS-RAF-MEK-ERK pathway. Specific mutations in *KRAS*, *NRAS*, and *BRAF*^V600E^ are classical examples that confer resistance to anti-EGFR therapy while also indicating targets for adjuvant therapies, as depicted in Figure 2 and Figure 3. Acquired mutations in the EGFR extracellular domain, such as the S492R mutation, demonstrate new resistance mechanisms that are rarely found and are therefore not routinely tested. However, they should be considered when previous alterations are absent. *HER2* and *MET* amplifications act as bypass mechanisms driving resistance to anti-EGFR antibodies. In addition, *PIK3CA* mutations and *PTEN* loss provide downstream activation independent of receptor blockade.

Amid the ongoing search for biomarkers that account for resistance mechanisms surpassing those attributable to *RAS* and *BRAF*, a spectrum of emerging molecular and microenvironmental biomarkers has been increasingly implicated in driving therapeutic failure [145] (Table 2, Figure 4). *MET* amplification is a known mechanism of acquired resistance to anti-EGFR therapy, occurring in a range of 2–18% of patients without *RAS*/*RAF* mutations nor microsatellite instability, depending on the detection methodology [146]. Mechanistically, *MET* activation bypasses EGFR blockade by sustaining downstream PI3K/AKT and MAPK signaling [147]. Importantly, recent data suggest that *MET* amplification can also be a primary (de novo) resistance factor in a small subset of *RAS* wild-type tumors [131]. Strategies combining EGFR inhibitors with MET inhibitors (e.g., crizotinib, capmatinib) have shown promise in patient-derived xenografts and early-phase clinical trials (e.g., involving molecularly selected cohorts) [148], suggesting that *MET* status should be part of the comprehensive genomic profiling for refractory mCRC.

Acquired mutations in the EGFR extracellular domain, such as the S492R mutation, can emerge during cetuximab therapy and prevent antibody binding, thereby causing resistance [149]. These are relatively rare and most relevant for cetuximab (not panitumumab). While not part of routine testing, the detection of an EGFR S492R mutation in ctDNA of a progressing patient would suggest that panitumumab could still be effective (since that mutation only interferes with cetuximab binding) [150,151]. This is a nuanced predictive scenario and an example of how specific resistance mutations can guide subsequent therapeutic choices in an iterative precision medicine approach.

Beyond purely genomic biomarkers, functional models are increasingly being explored to predict response and resistance to anti-EGFR therapies. For instance, patient-derived colorectal cancer organoids (PDOs) retain much of the genetic and phenotypic heterogeneity of the original tumors and can be exposed ex vivo to EGFR-targeted antibodies and antibody—drug conjugates (ADCs) to study both primary and acquired resistance mechanisms [152].

### 4.3. HER2 and Other Emerging Predictive Biomarkers

*HER2* (*ERBB2*) overexpression or amplification, well known in breast and gastric cancers, occurs in an important subset of CRC as well (~3–5% of mCRC overall), particularly in *RAS*/*BRAF* wild-type left-sided tumors [153]. *HER2* status is a predictive biomarker in two ways: (1) it confers resistance to anti-EGFR therapy, and (2) it identifies patients who may benefit from HER2-targeted treatments [154,155]. Retrospective analyses have found that *RAS*/*BRAF* wild-type mCRC patients whose tumors have *HER2* amplification do not respond well to cetuximab/panitumumab, presumably because HER2 activation provides an alternate growth signal [155]. Accordingly, clinical guidelines now recommend *HER2* testing in *RAS*/*BRAF* wild-type metastatic CRC to determine whether these patients should be steered away from EGFR inhibitors and receive HER2-targeted therapies instead [100,132]. Multiple phase II trials (HERACLES, MyPathway, Mountaineer) have shown the efficacy of dual HER2 blockade (e.g., trastuzumab + lapatinib, trastuzumab + pertuzumab) or newer HER2-directed agents (like trastuzumab-deruxtecan) in this subset, with response rates ~30–50% in heavily pretreated patients [156,157,158,159]. As a result, *HER2*-positive mCRC is now an actionable subtype, and trastuzumab with tucatinib (a HER2 kinase inhibitor) was recently FDA-approved for chemorefractory *RAS*/*BRAF* WT, *HER2*-amplified CRC [158].

Although extremely rare in CRC (<1% of cases), *NTRK1/2/3* gene fusions are actionable biomarkers that tend to occur in MSI-H cancers, specifically in the *RAS*/*BRAF* wild-type subset, or in those with certain histologies (e.g., secretory carcinoma) [160]. Tumors harboring these fusions are exquisitely sensitive to TRK inhibitor drugs (larotrectinib, entrectinib), with clinical trials demonstrating response rates > 70% across all *TRK* fusion-positive solid tumors, including CRC [111]. Accordingly, TRK inhibitors have received tissue-agnostic approval for any *TRK* fusion-positive cancer [161]. In CRC, routine *NTRK* screening is not cost-effective due to its rarity, but comprehensive genomic profiling may incidentally detect it, representing a strong indication for TRK inhibitor therapy in these cases.

A variety of other molecular alterations are being studied as potential predictive biomarkers. For example, *PIK3CA* mutations (in ~10–20% of CRCs) might predict a lack of benefit from EGFR inhibitors in *RAS* wild-type patients; however, current international guidelines consider the data inconsistent (likely differing by exon 9 vs. exon 20 mutations) and do not currently recommend them for routine exclusion of therapy outside clinical trials [132,162]. Tumors with *PTEN* loss or high *IGF2* expression have also been linked to EGFR inhibitor resistance [136]. A high tumor TGF-β signature or stromal gene expression (characteristic of the CMS4 subtype) may predict a lack of benefit from adjuvant chemotherapy in colon cancer [163], although this is still investigational. On the flip side, *BRAF* non-V600E mutations (e.g., D594G, G466R) appear to predict better outcomes and may be sensitive to anti-EGFR therapy, as these tumors behave more like *RAS* wild-type ones [164]. Tumor sidedness (right vs. left colon origin) is also recognized as a clinical predictive factor: patients with right-sided metastatic tumors generally derive minimal benefit from anti-EGFR antibodies, even if *RAS* wild-type, whereas left-sided tumors have a clear benefit [136]. Additionally, emerging data suggest that the gut microbiome might influence therapy response; specifically, presence of Fusobacterium in tumors has been associated with chemotherapy resistance in preclinical models [165] (Table 2), and efforts modulating the microbiome are underway to improve treatment efficacy.

### 4.4. Biomarkers for Therapeutic Response Monitoring

In addition to guiding initial treatment choice, biomarkers are increasingly used to monitor ongoing therapy and detect resistance or relapse at the earliest time point. Traditionally, imaging (CT scans) and clinical exam are used on therapy, but molecular biomarkers can sometimes signal treatment response or failure sooner.

Given the evidence that CEA monitoring can aid in detecting asymptomatic recurrences that may be amenable to curative intervention, serum levels are often monitored in patients with metastatic CRC receiving systemic therapy [132]. A decline in CEA levels over the first 1–2 months of chemotherapy often correlates with tumor response on scans, whereas a rise in CEA levels can foreshadow progression [166]. However, because CEA kinetics vary and some tumors do not produce CEA, this approach has only moderate sensitivity [167]. Still, CEA remains a simple and inexpensive adjunct to assess therapy effectiveness, and recommendation of checking CEA every 3–6 months for the first 2–3 years after surgery and then every 6 months up to 5 years is commonly included in most clinical guidelines [27]. Following this advice, marked CEA elevation should prompt re-evaluation in these cases, though confirmatory imaging is required due to a significant proportion of false positives.

ctDNA is not only a prognostic biomarker but is also now being used to guide therapy in clinical trials. The DYNAMIC trial demonstrated that a ctDNA-guided approach to adjuvant chemotherapy in stage II colon cancer could reduce overtreatment: patients who were ctDNA-negative were safely observed without chemotherapy (with low recurrence rates), whereas ctDNA-positive patients were treated (improving their outcomes) [168]. As noted, rising *RAS*-mutant allele fraction in blood can precede radiologic progression under EGFR therapy by a few months [136,169]. This raises the possibility of preemptive therapy switches: if a blood test shows a resistant clone emerging, treatment could be adjusted before clinical progression. To delve deeper into this aspect, ongoing trials, such as CIRCULATE and CHRONOS, are testing ctDNA-guided treatment adaptations [170,171]. A more cutting-edge approach is to measure ctDNA during treatment. Serial monitoring of ctDNA during follow-up can also provide an early warning of relapse (often months before imaging tests), helping to select patients who may be candidates for early intervention (e.g., salvage therapy) [11]. Conversely, rapid clearance of mutant ctDNA from plasma after starting therapy has been associated with better outcomes. In one study of mCRC patients on first-line chemotherapy, those who achieved undetectable ctDNA after two cycles had significantly longer progression-free survival than those with persistently detectable ctDNA [172].

Beyond ctDNA, circulating analytes like tumor-specific RNA are under investigation for post-treatment monitoring, and exosomal RNA as well as circulating tumor RNA (ctRNA) changes during therapy have also been correlated with therapeutic response in small studies [173,174].

Finally, tracking shifts in circulating tumor cell count or phenotype might signal changes in tumor biology, enabling treatment response surveillance and prompt emerging resistance detection [175]. For instance, a drop in CTC count after initiation of systemic therapy is generally a favorable sign, whereas rising CTCs can presage radiologic progression. In addition, an increase in mesenchymal-type CTCs can indicate an evolving EMT-driven resistance [176].

In summary, the integration of biomarkers into response monitoring holds great promise for personalizing therapy duration and sequence. For instance, a patient who achieves ctDNA clearance after a few cycles of chemotherapy might safely de-escalate treatment intensity, whereas one with rising ctDNA levels might need a change in regimen or early introduction of a new agent. Ultimately, these approaches aim to maximize efficacy while minimizing unnecessary toxicity, truly embodying precision medicine in real time.

## 5. Conclusions and Future Perspectives

The landscape of CRC biomarkers has expanded dramatically, spanning diagnostic, prognostic, and predictive applications, which are beginning to transform clinical practice. Established biomarkers such as *KRAS*/*NRAS* and *BRAF* mutations, MSI/dMMR status, and CEA are now cornerstones of personalized CRC management, guiding therapy choices and surveillance, while a wide range of emerging biomarkers—from DNA methylation panels and circulating miRNAs to Immunoscore and microbiome signatures—are moving toward translation. Nevertheless, clinical implementation is challenging: many promising biomarkers still require validation in large prospective trials to prove that their use improves outcomes, and issues of assay standardization (for example, across ctDNA platforms or IHC scoring systems), access, and cost-effectiveness must be addressed. In addition, the heterogeneous nature of CRC means that single biomarkers usually capture only a fraction of the underlying biology of the disease.

The integration of multi-omics data and advanced analytics is likely to underpin the next generation of biomarker development. Combining genomic, transcriptomic, epigenomic, proteomic, radiomic, and spatial-omics information can yield composite signatures that are more robust than any individual marker. Computational models that integrate mutation profiles, gene-expression subtypes, immune-cell infiltration metrics, and circulating biomarkers may provide comprehensive “molecular portraits” of individual tumors and more accurate predictions of risk and treatment response.

The use of biomarkers such as ctDNA to track tumor evolution and dynamically adjust treatment (“adaptive precision oncology”) is being explored in clinical studies and could shift practice from fixed lines of therapy toward more flexible strategies driven by early molecular signals of resistance or persistence. In parallel, non-invasive screening is expected to improve with next-generation stool and blood tests, including multi-target stool DNA assays and liquid-biopsy-based tests that incorporate microbial and metabolomic markers. Finally, deeper characterization of the tumor microenvironment and CRC immunobiology may yield new biomarkers that guide immunotherapies, particularly as these agents move into earlier lines of treatment and combination regimens.

Overall, the current landscape of CRC biomarkers is rich and rapidly evolving. Translating these advances into routine care will require coordinated efforts in terms of validation, standardization, and clinician and patient education. Yet the trajectory is clear: biomarker-driven approaches are poised to refine CRC screening, prognostication, and treatment. By tailoring interventions to the molecular features of each tumor and adapting those interventions as the tumor evolves over time, we move closer to truly personalized colorectal cancer care, with improved survival and quality of life as the ultimate goals.

## Figures and Tables

**Table 1:** Overview of diagnostic and screening biomarkers in CRC: traditional vs. emerging candidates.

Biomarker	Specimen	Method	Sensitivity/ Specificity	Clinical Use or Guideline Status
**Guaiac fecal occult blood test (gFOBT)**	Stool	Chemical reaction (peroxidase)	~70–80% (CRC) or ~60–80% (advanced adenoma)/~90%	Traditional screening (older); largely superseded by FIT due to diet confounders; still reduces CRC mortality.
**Fecal immunochemical test (FIT)**	Stool	Immunoassay (anti-hemoglobin Ab)	~79%/94%	First-line CRC screening (guideline-recommended in adults 45–75; e.g., USPSTF Grade A).
**Multitarget stool DNA (Cologuard)**	Stool	DNA assay (methylated BMP3/NDRG4 + KRAS + hemoglobin)	~92%/87%	FDA-approved (2014) for average-risk screening (3-year interval); endorsed as alternative to FIT by USPSTF/ACS.
**Plasma mSEPT9 (Epi proColon)**	Blood (plasma)	Methylation-specific PCR	~60–70%/~80–90%	FDA-approved (2016) for CRC screening (ages ≥ 45) but has inferior sensitivity; usually reserved for patients refusing other screening.
**Plasma ctDNA (Guardant Shield)**	Blood	NGS-based multi-gene cfDNA assay	~83%/90%	Newly FDA-approved (2024) multi-cancer CRC screen; not yet in routine guidelines; performance promising but evaluation ongoing.
**Carcinoembryonic antigen (CEA)**	Serum	Immunoassay	~50–70% (advanced CRC)/low in early stage	Not used for screening; guideline-recommended only for post-treatment surveillance (e.g., NCCN) (periodic monitoring for recurrence).
**Carbohydrate Ag19-9 (CA19-9)**	Serum	Immunoassay	<50% (CRC)	Not used for CRC screening or diagnosis; has limited role in advanced disease.
**Blood miRNA panel (e.g., miR-21, miR-92a)**	Blood	qRT-PCR/NGS	~80–90% (CRC, research studies)	Experimental—various panels reported high accuracy (e.g., stool miR panel with 88% CRC sensitivity); none validated or in guidelines.
**Stool miRNA panel (e.g., miR-21-5p, miR-199a-5p)**	Stool	qRT-PCR	~88% (CRC)/96% (CRC + advanced adenoma)	Investigational—shows promise in small studies, but not approved or recommended.

The “clinical use” column notes FDA approval or guideline recommendation status (e.g., USPSTF, ACS, NCCN), where applicable.

**Table 2:** Emerging biomarkers of resistance to anti-EGFR therapy in CRC.

Biomarker/Alteration	Mechanism of Resistance	Detection Technique	Clinical Relevance/Status
***MET* amplification**	Bypasses EGFR blockade via activation of parallel PI3K/AKT/MAPK signaling.	FISH/NGS (tissue or ctDNA)	Found in up to 18% of acquired resistance cases without *RAS*/*RAF* mutations nor microsatellite instability. Potential target for MET inhibitors (e.g., capmatinib) + anti-EGFR.
**EGFR ectodomain mutations (e.g., S492R)**	Prevents binding of specific monoclonal antibodies (e.g., cetuximab) to the receptor.	Liquid biopsy (ctDNA)/NGS	Mutations may prohibit cetuximab binding but allow panitumumab efficacy. Highlights the utility of liquid biopsy.
***HER2* amplification**	Heterodimerization with EGFR or independent signaling activation.	IHC/ISH/NGS	Predictive of anti-EGFR resistance. Actionable target with dual HER2 blockade (e.g., trastuzumab + lapatinib/tucatinib).
***NTRK* fusions**	Constitutive activation of TRK kinases driving tumor growth independent of EGFR.	IHC (pan-TRK)/NGS (RNA-seq)	Rare (<1%) but highly actionable with TRK inhibitors (larotrectinib, entrectinib).
***Fusobacterium nucleatum***	Modulates autophagy and immune microenvironment to support chemo/immunotherapy resistance.	qPCR (stool/tissue)/16S rRNA sequencing	High load correlates with recurrence and worse prognosis. Potential target for microbiome-modulating therapies.

## Data Availability

No new data were created or analyzed in this study. Data sharing is not applicable to this article.

## Abbreviations

The following abbreviations are used in this manuscript:

ADC	Antibody-drug conjugate
AI	Artificial intelligence
AUC	Area under the curve
CA19-9	Carbohydrate antigen 19-9
CEA	Carcinoembryonic antigen
cfDNA	Cell-free DNA
CMS	Consensus molecular subtype
CRC	Colorectal cancer
CTC	Circulating tumor cell
CSV	Cell-surface vimentin
ctDNA	Circulating tumor DNA
ctRNA	Circulating tumor RNA
dMMR	Deficient mismatch repair
EGFR	Epidermal growth factor receptor
ELISA	Enzyme-linked immunosorbent assay
EMT	Epithelial-to-mesenchymal transition
EV	Extracellular vesicle
FIT	Fecal immunochemical test
FOBT	Fecal occult blood test
HER2	Human epidermal growth factor receptor 2
HR	Hazard ratio
ICI	Immune checkpoint inhibitor
IHC	Immunohistochemistry
lncRNA	Long non-coding RNA
M2-PK	M2-pyruvate kinase
mCRC	Metastatic colorectal cancer
MMR	Mismatch repair
miRNA	MicroRNA
MRD	Minimal residual disease
MSI	Microsatellite instability
MSI-H	Microsatellite instability-high
MSS	Microsatellite stable
mt-sDNA	Multitarget stool DNA test
NCCN	National Comprehensive Cancer Network
NGS	Next-generation sequencing
NTRK	Neurotrophic tropomyosin receptor kinase
OS	Overall survival
PCR	Polymerase chain reaction
PFS	Progression-free survival
PDO	Patient-derived organoid
PD-1	Programmed death-1
PD-L1	Programmed death-ligand 1
qPCR	Quantitative polymerase chain reaction
TGF-β	Transforming growth factor beta
TILs	Tumor-infiltrating lymphocytes
TMB	Tumor mutational burden
TNM	Tumor-node-metastasis staging system
TRK	Tropomyosin receptor kinase
tsRNA	tRNA-derived small RNA
tRF	tRNA-derived fragment
VEGF	Vascular endothelial growth factor
